# Changing Epidemiology of Acute Respiratory Infections in Under-Two Children in Dhaka, Bangladesh

**DOI:** 10.3389/fped.2021.728382

**Published:** 2022-01-10

**Authors:** Karine Vidal, Shamima Sultana, Alberto Prieto Patron, Irene Salvi, Maya Shevlyakova, Francis Foata, Mahbubur Rahman, Iztiba Mallik Deeba, Harald Brüssow, Tahmeed Ahmed, Olga Sakwinska, Shafiqul Alam Sarker

**Affiliations:** ^1^Nestlé Institute of Health Sciences, Nestlé Research, Lausanne, Switzerland; ^2^International Center for Diarrheal Disease Research (icddr, b), Dhaka, Bangladesh; ^3^Department of Biosystems, Division of Animal and Health Engineering, University of Leuven, Leuven, Belgium

**Keywords:** acute respiratory infection, birth cohort, risk factors, infants, season

## Abstract

**Objectives:** Risk factors for acute respiratory infections (ARIs) in community settings are not fully understood, especially in low-income countries. We examined the incidence and risk factors associated with ARIs in under-two children from the Microbiota and Health study.

**Methods:** Children from a peri-urban area of Dhaka (Bangladesh) were followed from birth to 2 years of age by both active surveillance of ARIs and regular scheduled visits. Nasopharyngeal samples were collected during scheduled visits for detection of bacterial facultative respiratory pathogens. Information on socioeconomic, environmental, and household conditions, and mother and child characteristics were collected. A hierarchical modeling approach was used to identify proximate determinants of ARIs.

**Results:** Of 267 infants, 87.3% experienced at least one ARI episode during the first 2 years of life. The peak incidence of ARIs was 330 infections per 100 infant-years and occurred between 2 and 4 months of age. Season was the main risk factor (rainy monsoon season, incidence rate ratio [IRR] 2.43 [1.92–3.07]; cool dry winter, IRR 2.10 [1.65–2.67] compared with hot dry summer) in the first 2 years of life. In addition, during the first 6 months of life, young maternal age (<22 years; IRR 1.34 [1.01–1.77]) and low birth weight (<2,500 g; IRR 1.39 [1.03–1.89]) were associated with higher ARI incidence.

**Conclusions:** Reminiscent of industrialized settings, cool rainy season rather than socioeconomic and hygiene conditions was a major risk factor for ARIs in peri-urban Bangladesh. Understanding the causal links between seasonally variable factors such as temperature, humidity, crowding, diet, and ARIs will inform prevention measures.

## Introduction

Acute respiratory infections (ARIs) are a primary cause of morbidity worldwide (1) and a major cause of child mortality in the developing world. The predominant risk factors in previous studies conducted in less developed countries include poor sanitation, malnutrition, and exposure to indoor cooking smoke, which are typically linked to low socioeconomic and education status, especially in rural settings (2, 3). In high-income settings, the air quality related to parental smoking, and aspects linked to potential exposure to respiratory pathogens such as presence of siblings or day care attendance are often reported [e.g., (4, 5)]. Lack of, or short duration of, breastfeeding is the risk factor considered universally important (6, 7); the highest impact has been reported for low-resource settings, more severe infections (LRTI), and in youngest children (0–6 months) (8–10).

Interestingly, in countries such as Bangladesh that have successfully ameliorated mortality and morbidity due to diarrhea (11), the relative burden of ARIs has been growing (1). In this context, it could be hypothesized that the epidemiology and risk factors for ARIs may be rapidly changing and require re-examination.

Socioeconomic, maternal, and perinatal parameters are interrelated. It is a complex task to disentangle their direct and indirect effects on health outcomes. However, this understanding is crucial to devise the most efficient intervention strategies, especially in limited resource settings. For example, socioeconomic status may affect infant health outcomes through maternal nutritional status, health knowledge, or home sanitation. Although the alleviation of poverty is the optimal solution, shorter-term interventions to targeting, for example, home sanitation or maternal nutrition, differ. Only few studies directly addressed the interrelationship of these risk factors (4, 12), often due to incomplete record of potentially important variables.

The risk factors for ARIs during infancy are thought to vary depending on infant age. In early infancy (0–6 months), maternal diet and nutritional status could play an important role as they influence breast milk quality and output (13, 14) and limiting milk antibody transfer to infant (15). After 6 months, the child becomes more exposed to external environment and water, sanitation, and hygiene (WASH) as well as other environmental factors are thought to exert larger influence. However, comprehensive reports of age-specific risk factors in under-two children are infrequent.

We conducted a longitudinal, community-based study of ARIs in children followed from birth to 2 years living in Nandipara, a peri-urban community of Dhaka, Bangladesh (16). The goal of the present analysis is to examine a wide range of potential risk factors, including socioeconomic, environmental, WASH, and maternal and perinatal variables, employing an analytical hierarchical approach to identify the most likely causal links to ARI incidence.

## Methods

### Study Cohort

The Microbiota and Health Study (clinicaltrials.gov: NCT02361164) was a longitudinal, community-based cohort study conducted between April 2013 and October 2016 on 267 newborn infants born in Nandipara, a peri-urban community of Dhaka, Bangladesh, as previously described (16). Healthy pregnant women were enrolled in the third trimester of pregnancy. Gestational age was estimated by ultrasonography. The children were followed from birth to 2 years of age. Scheduled visits were done at 1 month of age and subsequently every 2 months during the first year of life, and then quarterly, at 15, 18, and 24 months of age. Infant anthropometric data and feeding practice were recorded at scheduled visits. Demographic, socioeconomic, and environmental characteristics were recorded during personal interview using an epidemiological questionnaire comparable with the Bangladesh Demographic and Health Survey.

### Acute Respiratory Infections

Active surveillance of ARI was conducted via weekly home visits in addition to the regular scheduled visits, by a community-based team of nurses supervised by a physician. Furthermore, mothers were asked to contact the study nurse whenever her infant experiences ARI symptoms. In such events, extra visits were performed at home or mother was asked to present the child to the health outpost. The diagnosis and the length of ARI were confirmed by the study medical officer. ARI diagnosis or symptoms were recorded as adverse events (AEs) with the starting and ending dates and documented in a standard electronic case report form by the study medical officer. In the absence of a specific diagnosis of ARI being recorded, ARI based on symptoms alone was defined as the sudden onset of ≥1 of the following symptoms, with or without fever: cough, runny nose, nasal congestion, ear discharge, and rapid breathing. A new ARI episode was defined as an episode starting after seven symptom-free days from the end of previous episode.

### Detection of Respiratory Pathogens

Nasopharyngeal samples were collected during the scheduled visits using flocked pediatric swabs as per manufacturer's instructions (Copan Diagnostics, Italy). The determination of three common facultative respiratory pathogens was performed at iccdr,b by bacterial culture using standard clinical diagnostics methods.

### Independent Variables

Demographic, maternal health, socioeconomic, and household characteristics collected at baseline, infants' characteristics at birth (16), as well as breastfeeding status and occurrence of ARIs in the first 2 months of life were considered as variables suspected to be associated with ARI burden. Selected categorical variables were recoded to obtain more balanced group sizes and to increase the power in the statistical analysis (Supplementary Table 1). A total of 35 variables were grouped into four categories: socioeconomic, environmental, perinatal, and postnatal (Table 1).

**Table 1:** Sociodemographic, environmental, prenatal, and postnatal characteristics of the 267 participants included in the Microbiota and Health study, Nandipara, Bangladesh, April 2013–October 2016.

**Level**	**Type of factor**	**Characteristic**	**Variable^**a**^**	**Category**	***n* (%)**
1	Socioeconomic	Household socioeconomic	Household income (Taka^b^ per month)^*1^	0: >10,000 1: ≥7,000 <10,000 2: <7,000	85 (31.8) 104 (39.0) 78 (29.2)
			House ownership^*6^	0: Yes 1: No	54 (24.5) 166 (75.5)
			Food insecurity^°^^*6^	0: No 1: Yes	194 (88.2) 26 (11.8)
			Mother's occupation^*1^	0: Housewife 1: Working (including student)	245 (91.8) 22 (8.2)
			Mother's education^*1^	0: Above primary 1: Primary 2: Illiterate	81 (30.3) 167 (62.5) 19 (7.1)
		Maternal characteristics	Mother's age (years) at screening^*1^	0: >22 1: ≤ 22	151 (56.6) 116 (43.4)
			Mother's height (cm) at screening^*2^	0: ≥150 1: <150	120 (45.1) 145 (54.9)
2	Environmental	Smoke exposure	Place of cooking^*6^	0: In the house 1: Outside the house	47 (21.4) 173 (78.6)
			Cooking fuel^*6^	0: Gas 1: Wood	209 (95.0) 11 (5.0)
			Smoker in family^*1^	0: No 1: Yes	89 (33.3) 178 (66.7)
		Household density	Number of people present at night per household^*6^	0: <4 1: ≥4	60 (27.3) 160 (72.7)
			Number of people present during the day^*6^	0: <4 1: ≥4	123 (55.9) 97 (44.1)
			Number of siblings^*3^	0: 0 1: ≥1	111 (42.0) 153 (58.0)
		Household structure	Flooring material^*6^	0: Cement 1: Wood 2: Earth	188 (85.5) 11 (5.0) 21 (9.5)
			Wall material^*6^	0: Tin 1: Solid walls, mud	123 (55.9) 97 (44.1)
		Drinking water indicator	Make water safer to drink^*6^	0: Yes 1: No	125 (56.8) 95 (43.2)
			Main source of drinking water^*6^	0: Safe 1: Less protected	201 (91.4) 19 (8.6)
			Location of drinking water^*6^	0: Dwelling 1: Yard, plot or elsewhere	24 (10.9) 196 (89.1)
		Sanitation	Toilet facility shared with other households^*6^	0: No 1: Yes	58 (26.4) 162 (73.6)
			Type of toilet facility^*6^	0: Improved 1: Unimproved	50 (22.7) 170 (77.3)
		Hygiene	Place of hand washing^*6^	0: In the house 1: Outside the house	35 (15.9) 185 (84.1)
			Hand washing^*6^	0: Improved 1: Unimproved	11 (5.0) 209 (95.0)
		Family history of respiratory illness	Person in family with chronic cough (>3 per year)^*1^	0: No 1: Yes	244 (91.4) 23 (8.6)
		Maternal reproductive	Gravida^*3^	0: No failed gravida 1: Failed gravida	184 (69.7) 80 (30.3)
			Mother's BMI (kg/m^2^) at screening^*2^	0: 18.5–25 1: <18.5 2: >25	150 (56.4) 93 (35.0) 23 (8.6)
			Blood group^*1^	0: A 1: AB 2: B 3: O	66 (24.7) 24 (9.0) 90 (33.7) 87 (32.6)
3	Perinatal	Delivery	Place of delivery^*1^	0: Hospital/clinic 1: Home	120 (44.9) 147 (55.1)
			Mode of delivery^*1^	0: Vaginal birth 1: C-section	199 (74.5) 68 (25.5)
			Season at birth^*1^	1. Pre-monsoon (MAR–MAY) 2. Rainy monsoon (JUN–OCT) 3. Cool dry winter (NOV–FEB)	89 (33.3) 115 (43.1) 63 (23.6)
		Infant characteristics	Gestational age at birth (weeks)^*1^	0: ≥37 (at term) 1: <37 (pre-term)	246 (92.1) 21 (7.9)
			Sex^*1^	0: Female 1: Male	140 (52.4) 127 (47.6)
			Weight at birth (kg)^*4^	0: >2.5 1: ≤ 2.5	185 (70.3) 78 (29.7)
4	Postnatal	Infant nutrition	Exclusively breastfed for ≥6 months^*5^	0: Yes 1: No	175 (71.4) 70 (28.6)
		History of illness	ARI within the first 2 months of infant life^*1^	0: No 1: Yes	220 (82.4) 47 (17.6)

a *Some of the variables were recoded to obtain more balanced group sizes and increase the power in the statistical analysis (see Supplementary Table 1)*.

b *7,000 taka corresponding to approximately 90 dollars or 390 international purchasing power parity (PPP) dollars between 2013 and 2016. °In the past 4 weeks, how often did you worry that your household would not have enough food?*

*Number of available data:

*1 *267 (100%)*,

*2 *266*,

*3 *264*

*4 *263*,

*5 *245*,

*and*

*6 *220*.

### Statistical Analysis

First, the identification of risk factors of ARI incidence was performed using negative binomial quasi-Poisson regression model, estimating unadjusted incidence risk ratios (IRR) and 95% CIs. In the next step, a previously described epidemiological conceptual framework (12) was adopted to identify the risk factors for ARI incidence. Briefly, variables were first grouped, considering the potential causal and temporal link to the outcome variable, here ARI incidence (Supplementary Figure 1). Starting from the most distal group (level 1), a multivariate regression including only the variables within this group was performed, estimating adjusted IRR. Any variables associated with ARI incidence at a level of *p* < 0.1 were included in the multivariate model for the next hierarchical level. Analysis was then repeated in a similar manner.

In addition, an exploratory analysis was conducted to explore the effects of time-variable factors, namely, breastfeeding status and nasopharyngeal bacterial pathogen colonization status. The outcome, ARI incidence, was analyzed using mixed-effects Poisson model, with the time-variable factors and variables that were significantly associated with ARIs in hierarchical analysis (age, ARI within the first 2 months of life, and season at episode) as fixed effects and child as random effect. All analyses were carried out using SAS (version 9.3).

### Ethics Statement

This study was approved by the Institutional Review Board (IRB) of icddr,b comprising Research Review Committee (RRC) and Ethical Review Committee (ERC). Study design, data collection, and baseline characteristics are provided elsewhere (16).

## Results

### Study Participants

Descriptive statistics of sociodemographic, environmental, prenatal, and postnatal characteristics of the participants included in the present analysis are shown in Table 1. Consistent with the 2014 survey from Bangladesh (17), exclusive breastfeeding was prevalent in our cohort: 71.4% of infants were exclusively breastfed and 26.5% were partially breastfed at the age of 6 months. Most infants continued to be partially breastfed to 24 months (Table 2). From the age of 6 months, infants received complementary foods usual for this population (Supplementary Table 2).

**Table 2:** Incidence of ARI episodes and number of children available for analysis.

	Breastfeeding status^*^ ( ***n*** **)**			**Incidence (ARI episodes per 100 IYs)**		
**Observation period**	**EBF**	**PBF**	**NBF**	**EBF**	**PBF**	**NBF**
0 to 2 months	248	5	1	85	120	600
2 to 4 months	213	26	4	315	208	450
4 to 6 months	173	64	5	333	169	240
6 to 8 months	1	227	7	0	222	429
8 to 10 months	0	229	7	N/A	160	171
10 to 12 months	0	223	6	N/A	145	100
12 to 14 months	0	217	7	N/A	105	0
15 to 17 months	0	215	9	N/A	64	0
18 to 20 months	0	206	9	N/A	61	0

* *Breastfeeding status during the 2 month observation period. EBF, exclusively breastfed; PBF, partially breastfed; NBF, not breastfed; N/A, not applicable*.

### ARI Incidence

**Figure 1:**
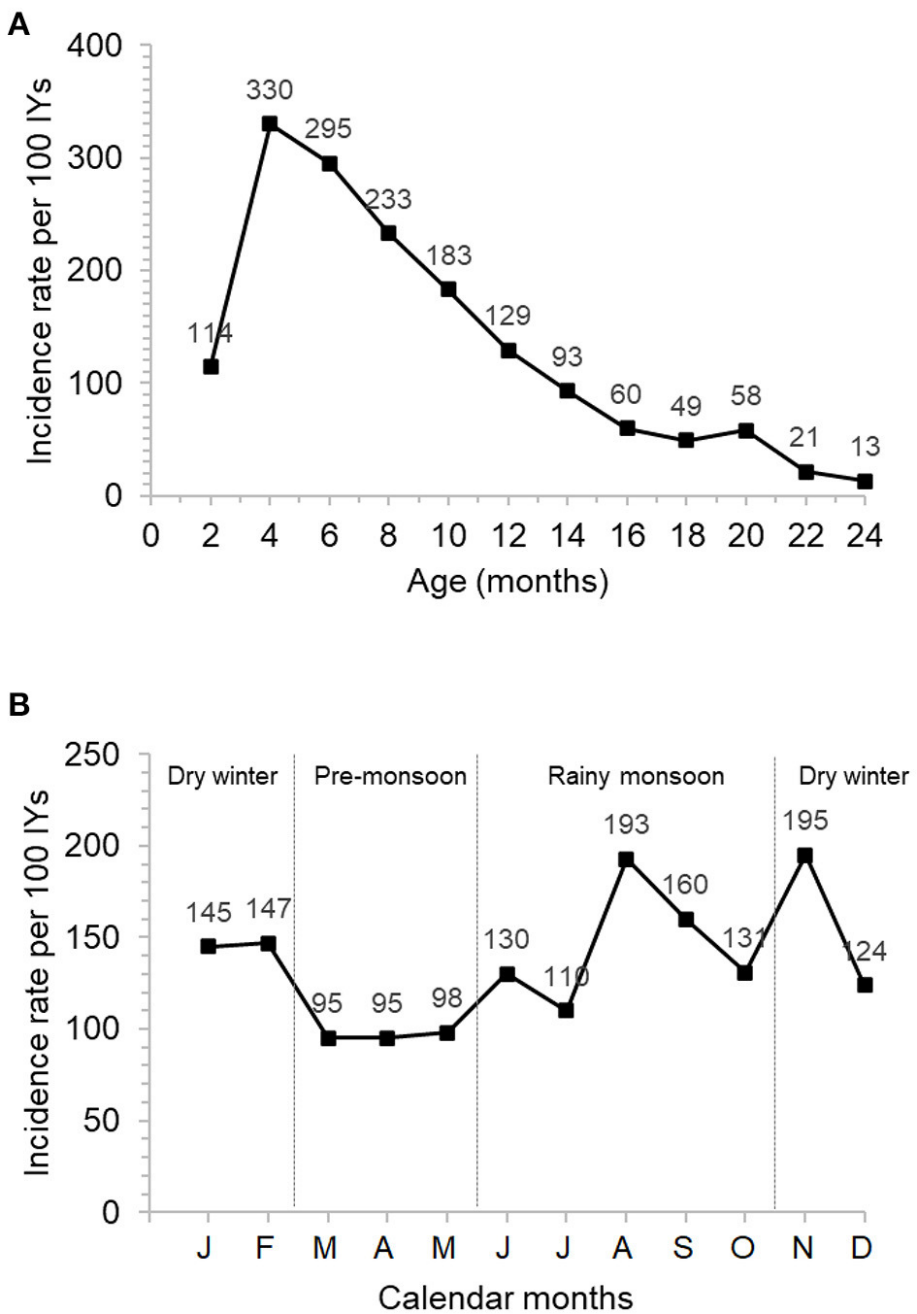
Incidence of ARI episodes by 2 month child age intervals **(A)** and by calendar months **(B)**.

In the first 2 years of life, a total of 665 episodes of ARIs were reported (Figure 1, Supplementary Figure 2A). Overall incidence was 136 ARI episodes per 100 infant-years (IYs) and average episode duration was 5.0 days (range: 2 to 16 days). The incidence was higher during the first than the second year of life (213 vs. 49 ARIs per 100 IYs, respectively), with a peak incidence of 330 ARIs per 100 IYs between 2 and 4 months of age (Figure 1A). Two seasonal peaks of ARIs were observed: one in August, in the middle of the rainy season (Jun–Oct), and one in November, at the beginning of the cool dry winter season (Nov–Feb) (Figure 1B). The most reported symptoms or diagnosis of ARIs were rhinorrhea and nasopharyngitis (Table 3, Supplementary Figure 3). Low number of acute otitis media cases were reported (Table 3; 7 and 9 in first and second year, respectively). Around 40% of the ARI episodes were associated with fever (Supplementary Figure 3). No cases of hospitalization associated to ARIs were reported during the study period. Most ARI episodes were treated with antihistaminic chlorpheniramine maleate (89%) (Supplementary Figure 2B) to relieve symptoms such as runny nose and cough. Prescription of antibiotics was mainly used to treat the otitis media and more severe ARIs (Table 3) and was rarely used in the first 2 months of life (4/50 ARI episodes) (Supplementary Figures 2C, 3).

**Table 3:** Manifestations of ARIs defined by symptom or diagnosis among children under 2 years of age.

**ARI symptom or diagnosis^**a**^**	**Number of ARI episodes**	**Prevalence *n* (%)**	**Incidence per 100 IYs**	**ARI symptom or diagnosis associated with either**		
				**Fever**	**Antibiotics**	**Anti-histamine use^**b**^**
Nasopharyngitis	267	148 (57.5)	54	110	22	245
Rhinorrhea	258	157 (62.7)	53	120	5	255
Cough	107	78 (30.0)	22	45	5	105
Nasal congestion	55	43 (16.4)	11	20	4	39
Otitis media	16	14 (5.7)	3	1	15	0
Unspecified	48	39 (15.7)	10	8	44	5

a *For ARI episodes defined by symptoms, episodes occurred alone or in combination with other symptoms. A total of 79 ARI episodes included more than one symptom of ARI. The ARI episodes defined by symptoms or specific diagnosis were recorded in the AE form and renamed using MedDRA preferred term. The terms reported in the AE form for nasopharyngitis were common cold and cold. The terms respiratory infection and upper respiratory tract infection were included in the category “unspecified.”*

b *Anti-histamine = chlorpheniramine maleate*.

### Risk Factors for ARI

**Figure 2:**
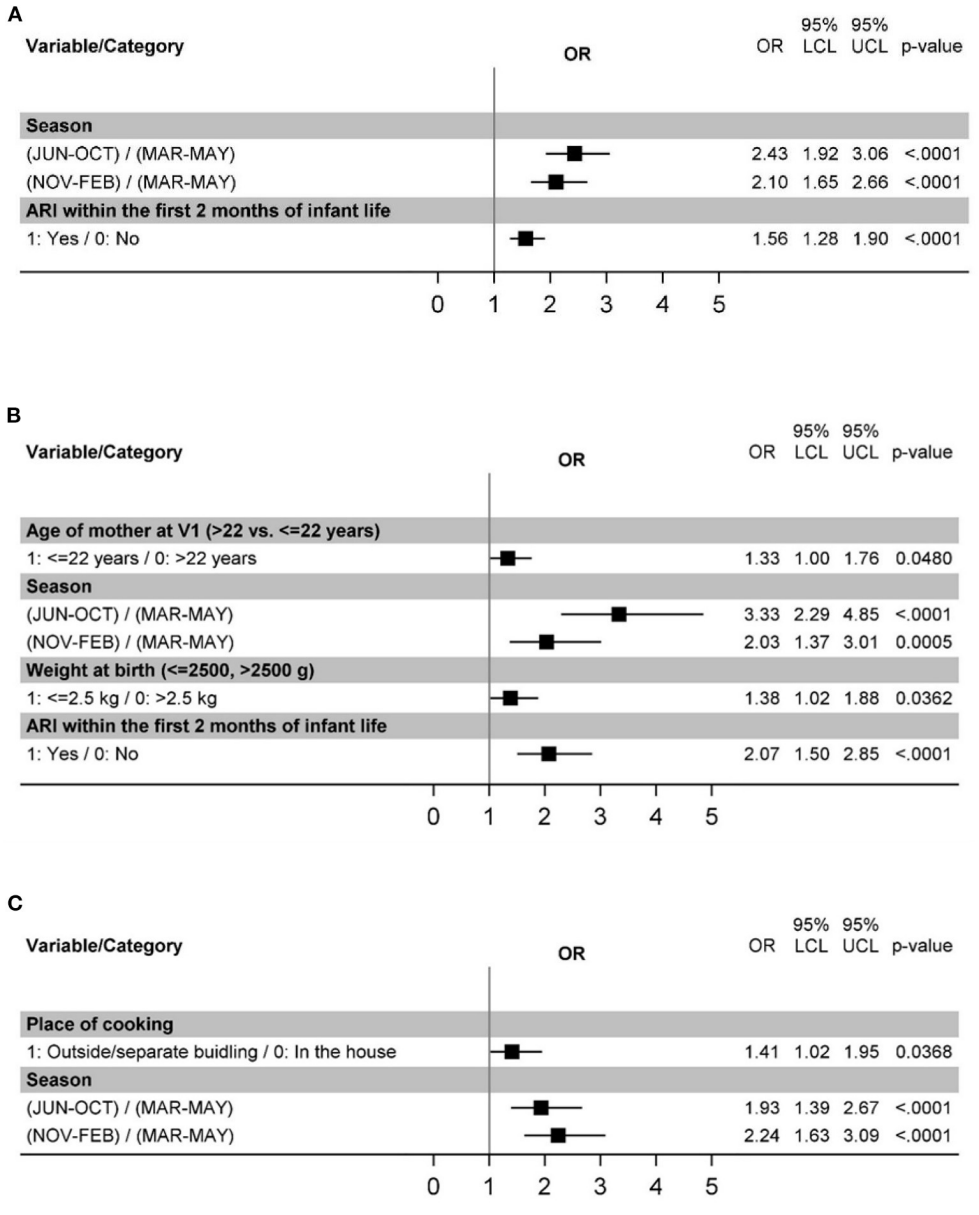
Risk factors for ARIs in the age intervals 0–24 months **(A)**, 0–6 months **(B)**, and 6–24 months **(C)**.

In the first 2 years of life, among all the variables examined (Table 1), the season (rainy monsoon, IRR 2.43 [1.92–3.06] or cool dry winter, IRR 2.10 [1.65–2.67] as compared with dry hot summer) and experiencing ARI episode in the first 2 months of life (IRR: 1.57 [1.28–1.90]) were associated with the ARI incidence in the hierarchical multivariate analysis (Table 4 and Figure 2).

**Table 4:** Variables associated with ARI incidence in children under 2 years of age.

**Level**	**Type of factors**	**Variable**	**Total^a^**	***n* (%)**	**IY**	**ARI episodes**	**Univariate analysis (p≤0.1)**		Multivariate analysis^*^	
							**IRR (95% CI)**	**p (Chisq)**	**IRR (95% CI)**	**p (Chisq)**
1	Socioeconomic	Mother education	267							
		0: Above primary		81 (30.3)	145.4	176				
		1: Primary		167 (62.5)	311.3	442	1.22 (0.98 to 1.51)	0.0725		
		2: Illiterate		19 (7.1)	34.5	47	*1.06 (0.83 to 1.36)*	*0.6216*		
2	Environmental	Place of cooking	220							
		0: Inside		47 (21.4)	94.3	106				
		1: Outside		173 (78.6)	343.7	495	1.27 (1.00 to 1.61)	0.0523	1.10 (0.84 to 1.44)	0.4698
		Place to wash hands	220							
		0: Inside		35 (15.9)	70.2	75				
		1: Outside		185 (84.1)	367.8	526	1.33 (1.01 to 1.75)	**0.0439**	1.15 (0.84 to 1.56)	0.3916
		Season								
		1. Pre-monsoon (MAR–MAY)			123.2	134				
		2. Rainy monsoon (JUN–OCT)			205.5	311	2.50 (1.98 to 3.14)	**<0.0001**	2.43 (1.92 to 3.06)	**<0.0001**
		3. Cool dry winter (NOV–FEB)			161.6	263	2.09 (1.65 to 2.65)	**<0.0001**	2.10 (1.65 to 2.66)	**<0.0001**
3	Perinatal	Place of delivery	267							
		0: Health facility		120 (44.9)	216.7	265				
		1: Home		147 (55.1)	274.6	400	1.23 (1.02 to 1.49)	**0.0333**	1.10 (0.92 to 1.31)	0.2992
4	Postnatal	Exclusively breastfed for ≥6 months	245							
		0: Yes		175 (71.4)	340.8	498				
		1: No		70 (28.6)	134.7	158	0.79 (0.65 to 0.97)	**0.0264**	0.84 (0.69 to 1.02)	0.0795
		ARI within the first 2 months of life	267							
		0: No		220 (82.4)	403.7	484				
		1: Yes		47 (17.6)	87.5	181	1.75 (1.41 to 2.17)	**<0.0001**	1.56 (1.28 to 1.90)	**<0.0001**

* *Hierarchical multivariate analysis of level 1 + level 2 + level 3 + level 4*.

a *Number of infants with available data. IY: infant-year; IR: incidence rate; IRR: incidence risk ratio; p (Chisq): p-value from χ^2^ test. In bold: significant p < 0.05*.

As we unexpectedly observed that the incidence of ARIs peaked before the age of 6 months, suggesting specific risk factors, exploratory analysis of episodes limited to the first 6 months of life was performed. This analysis confirmed the importance of the season (rainy monsoon, IRR 3.33 [2.29–4.85] or cool dry winter, IRR 2.03 [1.37–3.01] in contrast to hot dry summer; Figure 2B, Supplementary Table 3). ARI within the first 2 months of life were likewise significantly associated with ARI incidence (IRR 2.07 [1.50–2.85]) (Figure 2A, Supplementary Table 3). Additional risk factors identified for this age interval were young maternal age (<22 years, IRR 1.33 [1.00–1.76]) and low birth weight (IRR 1.38 [1.02–1.88]) Figure 2B, Supplementary Table 3).

Because the first ARI episodes occurred before the postnatal variables were observed and because experiencing an ARI episode in the first 2 months of life also contribute to the outcome variable of overall ARI incidence, an exploratory analysis of ARI episodes occurring between 6 and 24 months of age was carried out. When only the episodes occurring between 6 and 24 months of life were considered, season remained significantly associated with ARI incidence (rainy monsoon, IRR 1.93 [1.39–2.67] or cool dry winter, IRR 2.24 [1.63–3.09]; Figure 2C, Supplementary Table 4). In contrast, this was not observed for ARI within the first 2 months of life (Supplementary Table 4), where only a trend (*p*=0.074) was detected in univariate analysis. Outside household cooking place (IRR 1.41 [1.02–1.95]) was also identified as significant in this analysis (Figure 2C, Supplementary Table 4).

### Time-Variable Risk Factors

**Figure 3:**
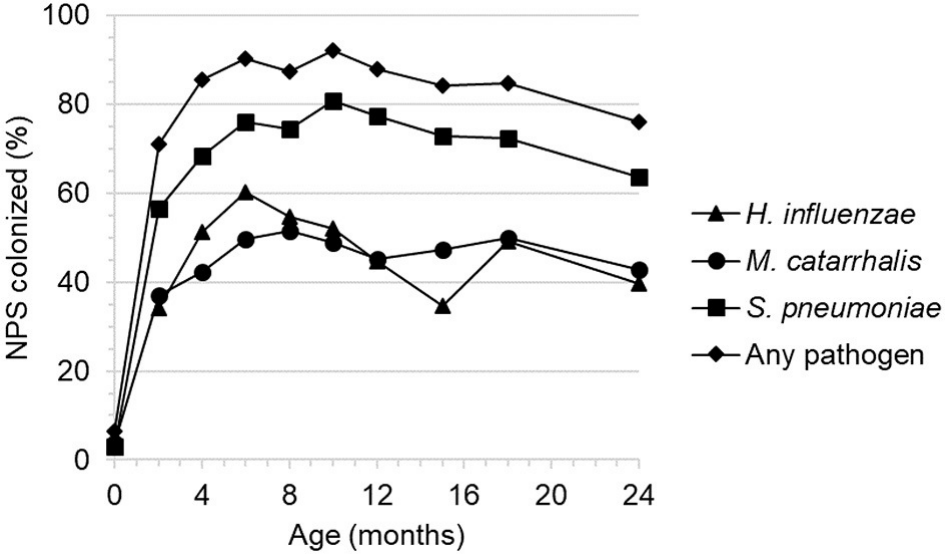
Nasopharyngeal colonization by three facultative respiratory bacterial pathogens by 2 month child age intervals.

Some of the expected significant risk modulators such as infant's breastfeeding status (Table 2) or nasopharyngeal colonization by respiratory bacterial pathogens (Figure 3) are dynamic features that vary throughout child age. We hypothesized that the association of such factors with ARIs could be best captured by a model associating breastfeeding status and colonization at a given time point with the incidence of ARIs in the subsequent 2 month period. This analysis included season at episode and occurrence of ARIs within the first 2 months of life, as these variables were associated with ARI risk in the multivariate hierarchical model. For the colonization status, only the data on infants who displayed no ARI symptoms during the sample collection were included.

The presence of three respiratory bacterial pathogens (*Haemophilus influenzae, Moraxella catarrhalis*, and *Streptococcus pneumoniae*) was evaluated by culture from infant nasopharyngeal samples collected at bi-monthly intervals (Figure 3). The level of colonization was low at birth, increased rapidly then plateaued by approximately 6 months of age and remained relatively high thereafter with a small decrease between 18 and 24 months.

Colonization by any of the three respiratory bacterial pathogens had a marginally significant association with ARIs occurring in the subsequent 2 month period (IRR 1.59 [1.13–2.24]) (Table 5). The risk appeared to be mainly linked to colonization by *M. catarrhalis* (IRR 1.48 [1.15–1.90]) (Supplementary Table 5). The lack of breastfeeding as compared with exclusive breastfeeding was significantly associated with increased incidence of ARIs in the concurrent time period (Table 5). Partial breastfeeding was also associated with higher ARI risk; however, the effect was weaker than for no breastfeeding. It is important to note that the number of non-breastfed infants was very low within this cohort (Table 2).

**Table 5:** Association between time-variable risk factors and ARI incidence within the subsequent 2 month period.

**Variable**	**Category**	**IRR (95% CI)**	***p***
Age	Age in months	1.17 (1.06 to 1.29)	0.0014
ARI within the first 2 months of life	0: No		
	1: Yes	1.97 (1.55 to 2.51)	<0.0001
Breastfeeding status^*^	0: EBF		
	1: NBF	5.60 (3.58 to 8.77)	<0.0001
	2: PBF	1.86 (1.17 to 2.94)	0.0083
Colonization status	0: No		
	1: Yes	1.59 (1.13 to 2.24)	0.0084
Season at episode	1. Pre-monsoon (MAR–MAY)		
	2. Rainy monsoon (JUN–OCT)	1.71 (1.29 to 2.27)	0.0002
	3. Cool dry winter (NOV–FEB)	1.21 (0.88 to 1.67)	0.2486

*The length of the period was chosen based on the availability of breastfeeding status data, recorded every 2 months. Model is adjusted for the interaction of age and breastfeeding status*.

* *Breastfeeding status during the 2 month observation period. EBF: exclusively breastfed; PBF: partially breastfed; NBF: not breastfed; IRR: incidence risk ratio*.

## Discussion

The present study examined multiple variables constituting potentially ARI risk factors in children under the age of 2 from the Microbiota and Health study, inhabiting Nandipara, a peri-urban community of Dhaka, Bangladesh, by employing hierarchical analytical framework assessment to identify the most relevant risk factors in this population.

Incidence of ARIs during the first 2 years of life observed in our study was 136 ARIs per 100 IYs, which is broadly within the range of other reports. Direct among-study comparisons are difficult due to the substantial effect of the study design (longitudinal population based, survey with short-term recall, and longitudinal cohort) on incidence estimates. The incidence of ARIs reached its peak between 2 and 4 months of age, which is considerably earlier than in other comparable studies from diverse settings (4, 5, 18, 19) where ARI incidence was highest around 12 months of age. Although no detailed age-interval breakdown is available in contemporary studies from Bangladesh (2, 20), ARI incidence in the first year of life is approximately 25% higher than in the second year. Together with the results of another early cohort study conducted in rural Bangladesh (21), and a more recent Indian study (22), these results suggest that the peak of ARIs tend to occur earlier in less developed countries.

Season was the most significant and consistent risk factor for ARI observed in our study. The incidence recorded during cool and rainy periods was 2- to 3-fold higher than in dry, warm months. Exploratory analysis indicated that this effect was the strongest among the youngest children, i.e., from birth to 6 months of age. Only few studies are designed to estimate the effect of the season; when it was examined, 2- to 3-fold higher incidence was revealed during cool winter in highly developed subtropical Australia (18), temperate Denmark (4), and in tropical rural Bangladesh (21). Multiple variables are linked to seasonal variation, such as the level of UV radiation, humidity, temperature, air pollution, effective indoor crowding, and diet. Identifying which of these factors are causally linked to ARIs is not trivial and requires large sample size. Disentangling the influence of these factors on ARI incidence would help to focus effective infection prevention strategies.

Our study indicated a previously appreciated protective effect of breastfeeding in regard to respiratory infections, while highlighting the limitations of standard analytical approaches. Dichotomizing the population into exclusively breastfed and not breastfed at the age of 6 months failed to reveal a significant effect on ARI risk; in contrast, time-dependent variable analysis indicated high incidence risk ratios related to partial and no breastfeeding as compared with exclusive breastfeeding. This inconsistency was likely due to the lack of robustness of the estimates, linked to imbalanced group size with non-breastfed infants constituting a very small proportion of study population (<5% of total numbers of infants, *n* = 10).

The occurrence of ARI before 2 months of age was associated with ARI incidence from birth to 2 years of life. However, this association was not detectable when the data on ARI incidence in the first 6 months of life were excluded, with only a weak trend present in univariate but not multivariate analysis. This suggests that the association represented a possible artifact due to partial inclusion of the predictor variable in the outcome variable, and that these very early episodes were neither a good predictor, nor a risk factor, for later infections.

We have observed a minor but significant association between colonization with respiratory bacterial pathogens and increased risk of subsequent ARIs, as suggested by earlier work (23). Most ARIs recorded in our study were brief (median of 5 days' duration) and relatively mild, necessitating treatment limited to symptom management. This suggests that bacterial etiology was somewhat unlikely. We hypothesize that increased colonization with bacterial pathogens may indicate immune status that influences susceptibility to viral infections, rather than solely being a marker of bacterial etiology. This may help explain similar, although somewhat weaker, incidence ratios observed when examining the associations separately for each pathogen species. The overall level of colonization appears to be relatively high, in particular in the first 6 months of life in comparison with reports from industrialized countries (24, 25).

Among perinatal variables, only low birth weight and young maternal age were weakly associated with increased ARI risk, and this effect was limited to the episodes occurring between birth and 6 months of age. While the effect of these and other perinatal factors such as gestational age or infant sex are often identified in large cross-sectional surveys (2, 3, 20, 26) conducted in less developed countries, longitudinal cohort studies from similar settings did not (21, 27), even if their sample size is similar. Cohort studies often include a relatively homogenous population that limits the detection of relatively subtle effects. The exclusion criteria used in cohort studies, such as enrollment late in pregnancy or exclusion of youngest mothers, likely diminish the power to detect by eliminating the high-risk individuals. In addition, associations with perinatal variables are generally more pronounced for more severe infections (LRTI) (4, 19, 28), while the majority of ARIs observed in this study were mild.

Among socioeconomic and WASH factors, only outside cooking place was significantly associated with ARI incidence for episodes occurring between 6 and 24 months, possibly reflecting the additional exposures these infants experienced when accompanying their mothers in the context of a community kitchen. A number of other variables showed weak associations (outside handwashing site, safety of drinking water) or trends (type of toilet facility and its sharing status among households) that were not significant in multivariate analysis. This may appear contradictory to previously observed robust associations between the ARI incidence, and socioeconomic and WASH factors in less developed countries (3, 26, 27, 29, 30). This apparent discrepancy could stem from the relatively homogenous socioeconomic status of participating families, as discussed previously in relation to perinatal variables. More interestingly, it is possible that our study population attained a developmental threshold where socioeconomic and WASH factors have ceased to play a major role. Maternal literacy rate was high (93%) and exposure to indoor smoke, known as an important ARI risk factor (31–33), was practically absent (95% of study households used natural gas as fuel as the infrastructure is available in the community). Among three economic and WASH factors (toilet type, type of fuel, wealth index) recorded in Bangladesh population surveys, all three were highly significantly associated with ARI (*p* < 0.0001) in 2004 (34), but only one (wealth index) remained so in 2014 (20).

This is reminiscent to observations from industrialized countries. For example, two recent studies (4, 35) examined multiple potential risk factors and both reported that season, child age, and childcare attendance were the only significant risk factors for respiratory infections, which is similar to our findings.

Our study has several strengths, such as longitudinal design in community settings, active surveillance of ARIs, collection of a wide range of variables, and hierarchical multivariate analysis, to assess the most relevant risk factors. The limitations include limited sample size precluding detection of more subtle effects and relatively homogenous population in respect to socioeconomic and environmental conditions. Even though effort were undertaken to record the most plausible risk factors, we did not, for example, separately capture maternal smoking, which may be gaining importance as an emerging risk factor in this and similar populations. The detection of specific etiology would offer additional insights, but the collection of samples during infectious episodes was beyond the scope of the study. Furthermore, the assessment of ARI severity was limited; hence, we focused our analyses on ARI incidence. Participating families relied on the study staff for primary medical care, potentially leading to limited morbidity or even incidence of ARIs compared with the general population.

In conclusion, we have found somewhat surprisingly that the most important determinants of ARIs in infants under 2 from the peri-urban community of Dhaka, Bangladesh resemble variables identified in industrialized settings, rather than socioeconomic and WASH factors. These data strongly suggest that infection prevention strategies need to adapt to changing epidemiology.

## Data Availability Statement

The raw data supporting the conclusions of this article will be made available by the authors, without undue reservation.

## Ethics Statement

The studies involving human participants were reviewed and approved by Research Review Committee and Ethical Review Committee, of International Centre for Diarrhoeal Disease Research, Bangladesh (ICDDR,B). Dhaka, Bangladesh. Written informed consent to participate in this study was provided by the participants' legal guardian/next of kin.

## Author Contributions

KV analyzed data and interpreted results and wrote the manuscript. SS coordinated and supervised data collection and reviewed and revised the manuscript. AP and IS interpreted the results and contributed to writing the manuscript with emphasis on the socioeconomic indicators. MS performed statistical analysis, interpreted results, and reviewed the manuscript. FF contributed to data management and analyses. MR designed and implemented study and analyzed data. ID supervised data collection. HB designed the study and reviewed the manuscript. TA designed and implemented the study and reviewed the manuscript. OS designed study, analyzed data, interpreted results, and wrote the manuscript. SS designed study, recruited participants, implemented study, and wrote the manuscript. All authors contributed to the article and approved the submitted version.

## Conflict of Interest

KV, AP, IS, MS, FF, HB, and OS are or were the employees of Société des Produits Nestlé S A at the time this work was performed. The remaining authors declare that the research was conducted in the absence of any commercial or financial relationships that could be construed as a potential conflict of interest.

## Publisher's Note

All claims expressed in this article are solely those of the authors and do not necessarily represent those of their affiliated organizations, or those of the publisher, the editors and the reviewers. Any product that may be evaluated in this article, or claim that may be made by its manufacturer, is not guaranteed or endorsed by the publisher.

## Abbreviations

ARI: Acute respiratory infection
EBF: Exclusively breastfed
IRR: Incidence rate ratio
IY: Infant-year
LRTI: Lower respiratory tract infection
NBF: Not breastfed
PBF: Partially breastfed
WASH: Water, sanitation and hygiene.
