# Supplementary material for: Changing Epidemiology of Acute Respiratory Infections in Under-Two Children in Dhaka, Bangladesh
Source: Front Pediatr. 2022 Jan 10;9:728382. doi: 10.3389/fped.2021.728382 (PMC8785242; doi:10.3389/fped.2021.728382)
Supplement: Supplementary file 1 [file Data_Sheet_1.docx]

Supplementary Material

## Supplementary Tables

**Supplementary Table 1**. List of variables recoded to obtain more balanced group sizes and increase the power in the statistical analysis.

| **Category** | **Original variables** | **Recoded variables** |
| --- | --- | --- |
| **Mother education** | Secondary | Above primary |
|  | High secondary |  |
|  | University |  |
|  | Primary | Primary |
|  | Illiterate | Illiterate |
| **Place of cooking** | In the house | Inside |
|  | In a separate building | Outside |
|  | Outdoors |  |
| **Food insecurity*** | Never | No |
|  | Often | Yes |
|  | Rarely |  |
|  | Sometimes |  |
| **Number of people present at night per household** | Adults present at night | <4 people |
|  | Children between 5- and 16-year old present at night |  |
|  | Children <5-year old present at night |  |
|  | Adults present at night | >=4 people |
|  | Children between 5- and 16-year old present at night |  |
|  | Children <5-year old present at night |  |
| **Number of people present during the day** | Adults present during the day | <4 people |
|  | Children between 5- and 16-year old present during the day |  |
|  | Children <5-year old present during the day |  |
|  | Adults present during the day | >=4 people |
|  | Children between 5- and 16-year old present during the day |  |
|  | Children <5-year old present during the day |  |
| **Water treatment prior to drinking** | Boiled | Make water safer to drink: Yes |
|  | Filtered |  |
|  | None | Make water safer to drink: No |
| **Main source of drinking water** | Piped into dwelling | Safe |
|  | Dug well protected |  |
|  | Tube well | Less protected |
|  | Piped to yard/plot |  |
|  | Dug well unprotected |  |
|  | Surface water |  |
| **Type of toilet facility** | Pit latrine with slab | Improved |
|  | Flush to piped sewer system | Unimproved |
|  | Flush to septic tank |  |
|  | Flush to pit latrine |  |
|  | Flush to somewhere else |  |
|  | Hanging toilet |  |
| **Hand washing** | Wash hands with soap after defecating | Improved |
|  | Wash hands after cleaning infant who defecated |  |
|  | Wash hands before feeding infant |  |
|  | Wash hands before preparing food |  |
|  | Wash hands before eating |  |
|  | No hand washing | Unimproved |
|  | Hand washing with water or with mud |  |

* In the past four weeks, how often did you worry that your household would not have enough food?

**Supplementary Table 2.** Complementary foods consumed, expressed as percentage of all infants for whom data were available.

| **Age (months)** | **Number of infants** | **Any food or drink besides breastmilk** | **Infant formula** | **Milk and milk drinks** | **Cereals, noodles or rice** | **Egg** | **Fish** | **Meat** | **Pulses** | **Vegetables** | **Fruit** | **Khichuri** | **Curry** |
| --- | --- | --- | --- | --- | --- | --- | --- | --- | --- | --- | --- | --- | --- |
| 1 | 267 | 2 | 2 | 0 | 0 | 0 | 0 | 0 | 0 | 0 | 0 | 0 | 0 |
| 2 | 263 | 3 | 3 | 0 | 0 | 0 | 0 | 0 | 0 | 0 | 0 | 0 | 0 |
| 4 | 252 | 12 | 12 | 1 | 2 | 0 | 0 | 0 | 0 | 0 | 0 | 0 | 0 |
| 6 | 247 | 29 | 21 | 8 | 22 | 0 | 0 | 0 | 0 | 0 | 0 | 2 | 0 |
| 8 | 246 | 99 | 31 | 22 | 92 | 11 | 6 | 1 | 5 | 10 | 4 | 64 | 0 |
| 10 | 238 | 100 | 24 | 19 | 97 | 36 | 30 | 7 | 21 | 36 | 8 | 54 | 0 |
| 12 | 235 | 100 | 15 | 22 | 100 | 68 | 73 | 29 | 54 | 80 | 17 | 15 | 3 |
| 15 | 231 | 100 | 11 | 15 | 100 | 77 | 87 | 46 | 66 | 89 | 19 | 10 | 6 |
| 18 | 229 | 100 | 8 | 20 | 100 | 85 | 88 | 59 | 79 | 93 | 26 | 4 | 3 |
| 24 | 220 | 100 | 3 | 35 | 100 | 93 | 96 | 75 | 94 | 94 | 41 | 2 | 1 |

**Supplementary Table 3.** Variables associated with ARI incidence in age interval 0 to 6 months.

| **Level** | **Type of factors** | **Variable** | **Total**^a^ | **n (%)** | **IY** | **ARI episodes** | **IR** | **Univariate analysis (p<=0.1)** | | **Multivariate analysis*** | |
| --- | --- | --- | --- | --- | --- | --- | --- | --- | --- | --- | --- |
|  |  |  |  |  |  |  |  | **IRR (95%CI)** | **p (Chisq)** | **IRR (95%CI)** | **p (Chisq)** |
| **1** | **Maternal** | Age of mother at screening | 267 |  |  |  |  |  |  |  |  |
|  |  | 0: >22 years |  | 151 (56.6) | 75.3 | 161 | 214 |  |  |  |  |
|  |  | 1: <=22 years |  | 116 (43.4) | 55.7 | 158 | 284 | 1.28 (1.01 to 1.61) | **0.0373** | 1.33 (1.00 to 1.76) | **0.0480** |
| **2** | **Environmental** | Make water safer to drink | 220 |  |  |  |  |  |  |  |  |
|  |  | 0: Improved |  | 125 (56.8) | 62.5 | 140 | 224 |  |  |  |  |
|  |  | 1: Unimproved |  | 95 (43.2) | 47.5 | 140 | 295 | 1.32 (1.03 to 1.67) | **0.0254** | 1.15 (0.86 to 1.55) | 0.3479 |
|  |  | Toilet facility shared with other households | 220 |  |  |  |  |  |  |  |  |
|  |  | 0: No |  | 58 (26.4) | 29.0 | 88 | 303 |  |  |  |  |
|  |  | 1: Yes |  | 162 (73.6) | 81.0 | 192 | 237 | 0.78 (0.60 to 1.01) | 0.0634 | 0.80 (0.58 to 1.11) | 0.1859 |
|  |  | Type of toilet facility | 220 |  |  |  |  |  |  |  |  |
|  |  | 0: Improved |  | 50 (22.7) | 25.0 | 76 | 304 |  |  |  |  |
|  |  | 1: Unimproved |  | 170 (77.3) | 85.0 | 204 | 240 | 0.79 (0.60 to 1.04) | 0.0894 | 0.99 (0.71 to 1.37) | 0.9354 |
|  |  | Place to wash hands | 220 |  |  |  |  |  |  |  |  |
|  |  | 0: Inside |  | 35 (15.9) | 17.5 | 31 | 177 |  |  |  |  |
|  |  | 1: Outside |  | 185 (84.1) | 92.5 | 249 | 269 | 1.52 (1.04 to 2.22) | **0.0313** | 1.42 (0.89 to 2.28) | 0.1391 |
|  |  | Season |  |  |  |  |  |  |  |  |  |
|  |  | 1. Pre-monsoon |  |  | 26.9 | 55 | 204 |  |  |  |  |
|  |  | 2. Rainy monsoon |  |  | 67.8 | 170 | 251 | 3.25 (2.29 to 4.62) | **<0.0001** | 3.33 (2.29 to 4.85) | **<0.0001** |
|  |  | 3. Cool dry winter |  |  | 36.2 | 102 | 281 | 1.88 (1.30 to 2.74) | **0.0009** | 2.03 (1.37 to 3.01) | **0.0005** |
| **3** | **Perinatal** | Place of delivery | 267 |  |  |  |  |  |  |  |  |
|  |  | 0: Health facility |  | 120 (44.9) | 58.9 | 128 | 217 |  |  |  |  |
|  |  | 1: Home |  | 147 (55.1) | 72.1 | 191 | 265 | 1.22 (0.96 to 1.54) | 0.1003 | 1.02 (0.76 to 1.37) | 0.892 |
|  |  | Weight at birth | 263 |  |  |  |  |  |  |  |  |
|  |  | 0: >2.5 kg |  | 185 (70.3) | 90.7 | 202 | 223 |  |  |  |  |
|  |  | 1: <=2.5 kg |  | 78 (29.7) | 38.3 | 113 | 295 | 1.33 (1.04 to 1.69) | **0.0217** | 1.38 (1.02 to 1.88) | **0.0362** |
| **4** | **Postnatal** | Exclusively breastfed for >=6 months | 245 |  |  |  |  |  |  |  |  |
|  |  | 0: Yes |  | 175 (71.4) | 89.5 | 234 | 278 |  |  |  |  |
|  |  | 1: No |  | 70 (28.6) | 35.0 | 68 | 194 | 0.70 (0.53 to 0.92) | **0.0100** | 0.77 (0.55 to 1.07) | 0.1241 |
|  |  | ARI within the first 2 months of life | 267 |  |  |  |  |  |  |  |  |
|  |  | 0: No |  | 220 (82.4) | 107.8 | 215 | 199 |  |  |  |  |
|  |  | 1: Yes |  | 47 (17.6) | 23.2 | 104 | 449 | 2.26 (1.79 to 2.86) | **<0.0001** | 2.07 (1.50 to 2.85) | **<0.0001** |

^a^: Number of infants with available data. Percentages are calculated on the basis of available frequencies. IY: Infant-Year. IR: Incidence rate. IRR: Incidence Risk Ratio; CI: Confidence Interval; p (Chisq): p-value from Chis square test; *Hierarchical multivariate analysis of Level 1 + Level 2 + Level 3 + Level 4; In bold: significant p-value <0.05.

**Supplementary Table 4.** Variables associated with ARI incidence in age interval 6 to 24 months.

| **Level** | **Type of factors** | **Variable** | **Total**^a^ | **n(%)** | **IY** | **ARI episodes** | **IR** | **Univariate analysis (p<=0.1)** | | **Multivariate analysis*** | |
| --- | --- | --- | --- | --- | --- | --- | --- | --- | --- | --- | --- |
|  |  |  |  |  |  |  |  | **IRR (95%CI)** | **p (Chisq)** | **IRR (95%CI)** | **p (Chisq)** |
| **2** | **Environmental** | Place of cooking | 220 |  |  |  |  |  |  |  |  |
|  |  | 0: Inside |  | 47 (21.4) | 70.5 | 51 | 72 |  |  |  |  |
|  |  | 1: Outside |  | 173 (78.6) | 256.6 | 270 | 105 | 1.44 (1.03 to 2.01) | **0.0332** | 1.41 (1.02 to 1.95) | **0.0368** |
|  |  | Season |  |  |  |  |  |  |  |  |  |
|  |  | 1. Pre-monsoon (MAR-MAY) |  |  | 96.7 | 79 | 82 |  |  |  |  |
|  |  | 2. Rainy monsoon (JUN-OCT) |  |  | 136.7 | 141 | 103 | 1.91 (1.39 to 2.63) | **<0.0001** | 1.93 (1.39 to 2.67) | **< 0.0001** |
|  |  | 3. Cool dry winter (NOV-FEB) |  |  | 126 | 161 | 128 | 2.25 (1.65 to 3.08) | **<0.0001** | 2.24 (1.63 to 3.09) | **< 0.0001** |
| **3** | **Perinatal** | Place of delivery | 267 |  |  |  |  |  |  |  |  |
|  |  | 0: Health facility |  | 120 (44.9) | 157.2 | 137 | 87 |  |  |  |  |
|  |  | 1: Home |  | 147 (55.1) | 202.1 | 209 | 103 | 1.25 (0.97 to 1.60) | 0.0864 | 1.11 (0.87 to 1.42) | 0.3957 |
| **4** | **Postnatal** | ARI within the first 2 months of life | 267 |  |  |  |  |  |  |  |  |
|  |  | 0: No |  | 220 (82.4) | 295.1 | 269 | 91 |  |  |  |  |
|  |  | 1: Yes |  | 47 (17.6) | 64.2 | 77 | 120 | 1.32 (0.97 to 1.79) | 0.0743 | 1.18 (0.88 to 1.59) | 0.2682 |

^a^: Number of infants with available data. Percentages are calculated on the basis of available frequencies. IY: Infant-Year. IR: Incidence rate. IRR: Incidence Risk Ratio; CI: Confidence Interval; p (Chisq): p-value from Chis square test; *Hierarchical multivariate analysis of Level 1 + Level 2 + Level 3 + Level 4; In bold: significant p-value <0.05.

**Supplementary Table 5.** Association between time-variable risk factors and ARIs incidence occurring in the subsequent two-month period. Model is adjusted for the interaction of age and breastfeeding status. Colonization status indicates the presence of one bacteria species assessed independently.

| **Variable** | **Category** | **IRR (95% CI)** | **p** |
| --- | --- | --- | --- |
| ***H. influenzae*** |  |  |  |
| Age | Increment by 30 days | 1.26 (1.17 to 1.36) | <0.0001 |
| ARI in the first 2 months | 0: No |  |  |
|  | 1: Yes | 1.95 (1.52 to 2.49) | <0.0001 |
| Breastfeeding status* | 0: EBF |  |  |
|  | 1: NBF | 6.39 (3.84 to 10.65) | <0.0001 |
|  | 2: PBF | 2.32 (1.56 to 3.47) | <0.0001 |
| Colonization status | 0: No |  |  |
|  | 1: Yes | 1.09 (0.85 to 1.39) | 0.5051 |
| Season at episode | 1. Pre-monsoon (MAR-MAY) |  |  |
|  | 2. Rainy monsoon (JUN-OCT) | 1.65 (1.24 to 2.19) | 0.0005 |
|  | 3. Cool dry winter (NOV-FEB) | 1.22 (0.88 to 1.69) | 0.2379 |
| ***M. catarrhalis*** |  |  |  |
| Age | Increment by 30 days | 1.24 (1.15 to 1.34) | <0.0001 |
| ARI in the first 2 months | 0: No |  |  |
|  | 1: Yes | 1.93 (1.51 to 2.46) | <0.0001 |
| Breastfeeding status* | 0: EBF |  |  |
|  | 1: NBF | 6.20 (3.89 to 9.90) | <0.0001 |
|  | 2: PBF | 2.19 (1.46 to 3.29) | 0.0001 |
| Colonization status | 0: No |  |  |
|  | 1: Yes | 1.48 (1.15 to 1.90) | 0.0022 |
| Season at episode | 1. Pre-monsoon (MAR-MAY) |  |  |
|  | 2. Rainy monsoon (JUN-OCT) | 1.80 (1.35 to 2.41) | <0.0001 |
|  | 3. Cool dry winter (NOV-FEB) | 1.17 (0.84 to 1.61) | 0.3544 |
| ***S. pneumoniae*** |  |  |  |
| Age | Increment by 30 days | 1.24 (1.14 to 1.35) | <0.0001 |
| ARI in the first 2 months | 0: No |  |  |
|  | 1: Yes | 1.97 (1.54 to 2.51) | <0.0001 |
| Breastfeeding status* | 0: EBF |  |  |
|  | 1: NBF | 6.28 (3.91 to 10.10) | <0.0001 |
|  | 2: PBF | 2.15 (1.41 to 3.28) | 0.0004 |
| Colonization status | 0: No |  |  |
|  | 1: Yes | 1.24 (0.94 to 1.63) | 0.1275 |
| Season at episode | 1. Pre-monsoon (MAR-MAY) |  |  |
|  | 2. Rainy monsoon (JUN-OCT) | 1.68 (1.26 to 2.24) | 0.0004 |
|  | 3. Cool dry winter (NOV-FEB) | 1.22 (0.88 to 1.70) | 0.2273 |

*Breastfeeding status during the two-months observation period. EBF: exclusively breastfed; PBF: partially breastfed; NBF: not breastfed. IRR: Incidence Risk Ratio; CI: Confidence Interval.

## Supplementary Figures


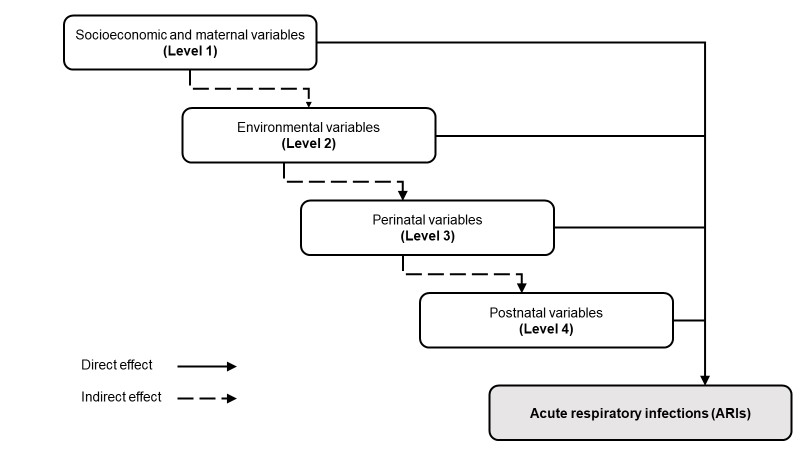


**Supplementary Figure 1.** Analytical conceptual framework.


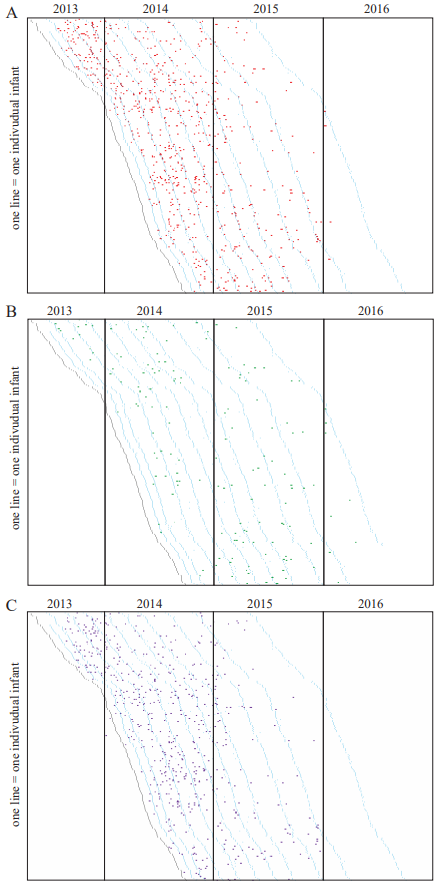


**Supplementary Figure 2.** Visualization of ARI episodes (A), antihistamine chlorpheniramine maleate administration (B) and antibiotic treatment (C) occurring per infant during the whole study, April 2013-October 2016. Each line represents a subject with: The light green/orange dots representing the birthdate of each infant, and the blue dots represent the regular scheduled visits (e.g. 1, 2, 4, 6 months). The vertical lines delimit the different years of the study.


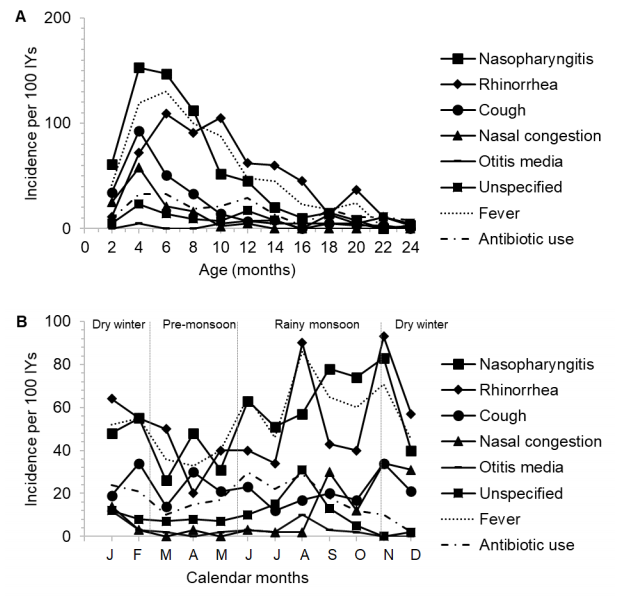


**Supplementary Figure 3.** Incidence of ARI episodes defined by symptom or diagnosis, associated with fever or antibiotic treatment, by two-month child age intervals (A) and by calendar months (B). ARI symptoms or specific diagnosis were recorded in the AE form and renamed using MedDRA preferred term. The terms reported in the AE form for nasopharyngitis were common cold and cold. The terms respiratory infection and upper respiratory tract infection were included in the category “unspecified”.


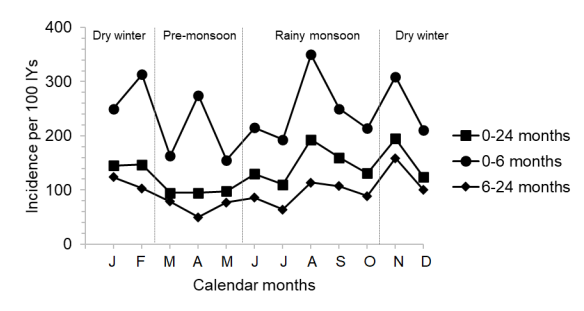


**Supplementary Figure 4.** Incidence of ARI episodes by calendar month for episodes occurring at different age intervals.
